# Modeling Challenge Data to Quantify Endogenous Lactate Production

**DOI:** 10.3389/fendo.2021.656054

**Published:** 2021-06-28

**Authors:** Darko Stefanovski, Pamela A. Wilkins, Raymond C. Boston

**Affiliations:** ^1^ Department of Clinical Studies – New Bolton Center, School of Veterinary Medicine, University of Pennsylvania, Kennett Square, PA, United States; ^2^ Department of Veterinary Clinical Medicine, College of Veterinary Medicine, University of Illinois, Urbana-Champaign, IL, United States; ^3^ Department of Clinical Studies- New Bolton Center, School of Veterinary Medicine, University of Pennsylvania, Kennett Square, PA, United States

**Keywords:** lactate, mathematical model, horse, metabolism, infusion, units

## Abstract

With the intention of isolating the susceptibility of modeling methodology to influence our investigation of the infusion data, we used three kinetic approaches to our models: a simple approach, a unit approach, and a novel approach. The simple approach used exclusively built-in modeling features of the software in terms of units of the infusion dilution (mmol/L), as well as in terms of the precision of switching the infusion on and off. The unit approach used the same switching mechanism as the simple approach, but the units were modeled in those of the infusion (e.g., mmol/kg). Thirdly with the novel approach, we used an automated approach to controlling the infusion, in the sense that as the modeling mechanism sensed the slowdown of the infusion, it was gradually turned off. The units of the analysis for the novel approach were exactly the same as those deployed in the unit approach. Our objective here was to see if common pharmacokinetic parameters were seriously impacted by the particular modeling method.

## Introduction

Recent times have seen a rapid expansion in the use of challenge studies to help quantitate endogenous production of metabolites. The reasons for this are clear: unlike steady-state investigations, they are not as time costly as there is no need to equilibrate multiple analytes by leveraging others (e.g., establishing a new steady state for glucose while infusing insulin) that often takes several hours, the results are relatively easy to interpret, and they need not necessarily focus kinetically to beyond the specific metabolite of interest. L-lactate (LAC) is one such key cellular metabolite, produced by every cell and oxidized by those containing mitochondria; its metabolism is central to energy homeostasis and the cellular redox state. LAC has both beneficial and even essential functions in several metabolic disorders (1–4).

LAC is well recognized as a prognostic indicator in many severe disease states, both in humans and animals, and it is not necessarily a detrimental factor. Clinically, initially, in many disease states, aberrations in circulating LAC concentrations (blood LAC) are assumed to result from perfusion disturbances, resulting in increased production. Changes in blood LAC in later stages of diseases such as sepsis are thought to result from continued increased production, aberrant metabolism, including decreases in elimination, or both. While the majority of LAC produced by the body is metabolized in the liver (converted back to glucose and then stored as glycogen), 20-30% is removed by the kidney (5). Of this, only 10-12% is thought to be eliminated *via* urinary excretion, the rest removed by uptake and metabolism within the kidney (6).

Single or serial measurement of blood [LAC] is considered a reliable prognostic indicator in critically ill foals and adult horses (7–11). Endogenous LAC clearance has been similarly used, relying on various techniques employing changes in [LAC] over time (10–18). The various estimates of lactate ‘clearance’ (decrease or disappearance from the blood) used in earlier studies suggested that estimates of [LAC] ‘clearance’ is more useful than single measurements of [LAC] (10, 12, 15, 19). Calculation of true clearance of exogenously administered L-lactate (ExLC) in hemodynamically stable septic human patients was shown in 2 studies to be a useful prognostic indicator (4, 13). The technique allowed for a determination of true clearance -in addition to the production of LAC- with utility in interrogating the underlying processes of hyperlactatemia in critically ill human and veterinary patients. An equine species-specific ExLC test has been developed for use in horses (20).

The specific aim of this report is, using a study, and data, outlined earlier (20), to introduce an array of approaches enabling us to readily characterize the disposition of lactate subsequent to a brief infusion. We will explore how a selection of the units in which the infusion is modeled and the modeling approach per se (e.g., modeling elements used) to portray the infusion itself, and each may have evident consequences in our investigation and the interpretation of an infusion-based challenge to a system. As a consequence of our novel modeling approaches described here, we will capitalize on the WinSAAM software (www.winsaam.org) (21) and we will use the opportunity here to explain some less well-known features of this computational tool for compartmental analysis.

## Materials and Methods

Institutional approval: All procedures were approved by the Institutional Animal Care and Use Committee.

**Figure 1:**
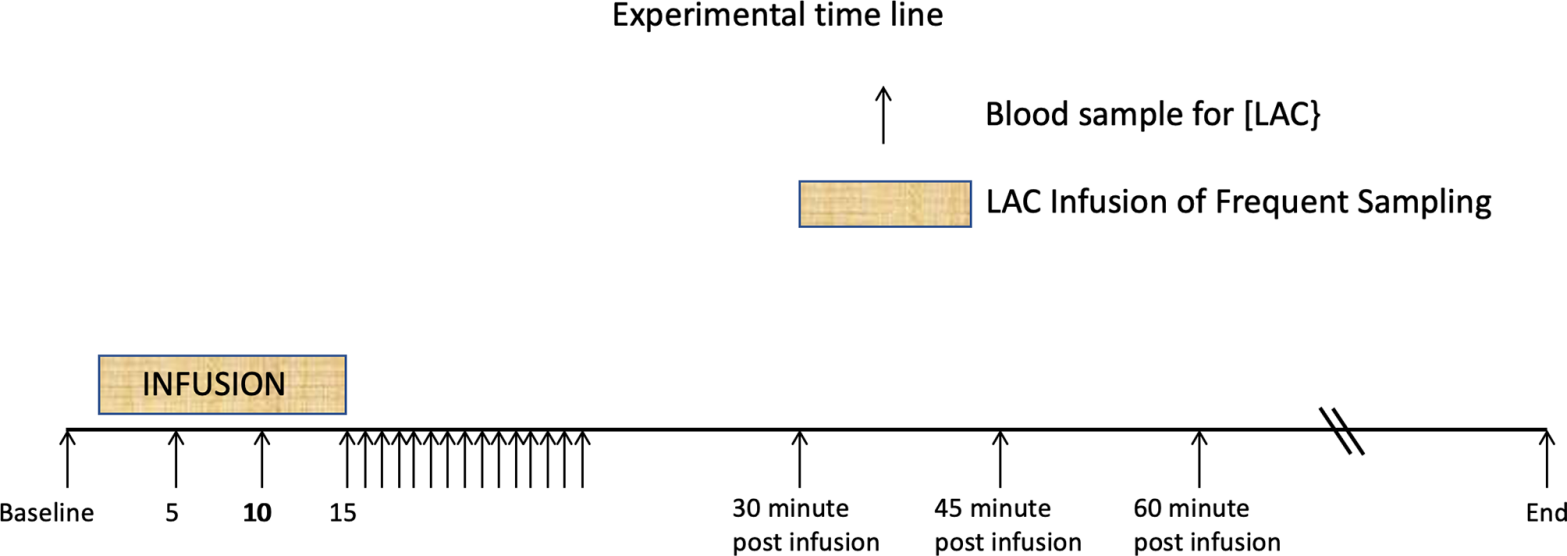
Time line showing 15 minute periods one of L-Lactate infusion, and the other of rapid sampling. These are followed by the postinfusion sampling strategies. Additional samples were drawn until the baseline lactate was reached. From Figure 1 from P De Pedro et al. J. Vet. Emerg. Crit. Care 22 (5)2012. pp 564-572 (20).

L-lactate infusion: The LAC infusion protocol has been described in detail elsewhere (20). Briefly, 500 ml sterile 0.9% NaCl solutions containing 1 mmol.kg^-1^ body weight of lactate were infused into the jugular vein of 5 healthy adult horses using an infusion pump over 15 min. The opposite jugular vein was sampled at various times, with blood [LAC] determined using a YSI lactate meter (**Figure 1**). No other analytes such as glucose, insulin or triglycerides were collected during this experimental protocol.

### Pattern of Lactate Disposition

**Figure 2:**
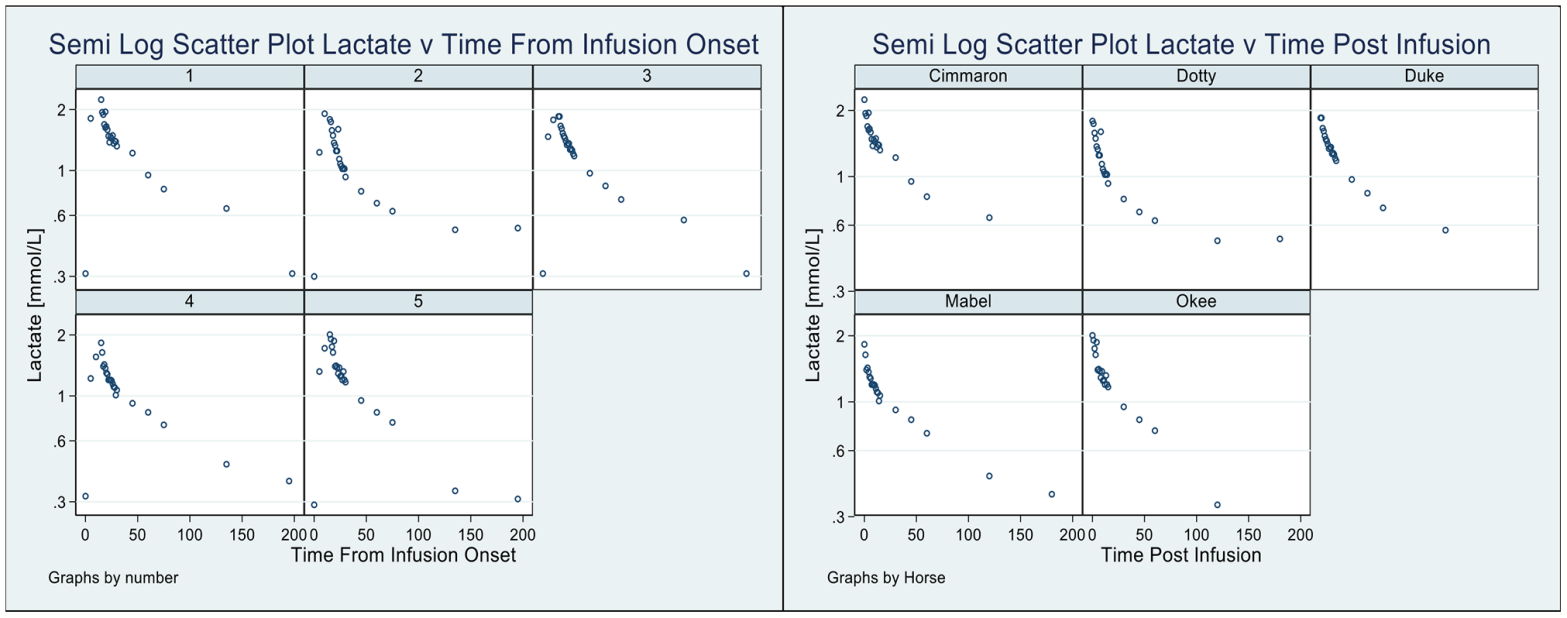
Pattern of lactate disposition, by horse, from sample immediately prior to infusion to the final observation (Left), and also shown (Right) the pattern of lactate disposition from the completion of the infusion to the final observation.

In **Figure 2**, using classical exploratory methods (22), we present two perspectives of the lactate disposition: the pattern of lactate (for each horse) from the time immediately prior to the lactate infusion (**Figure 2**, left) until the lactate level returned to its value prior to the infusion; and, the pattern of lactate (**Figure 2**, right, again for each horse) from the cessation of the infusion until the lactate returned to its mean baseline value (20). Three features of these graphs are as follows: 1) in both cases there is considerable variation in aspects of the disposition, 2) the mean baseline lactate value is slightly higher than the lactate value just prior to the infusion, and 3) a semilog pattern, evident in each graph, is strongly suggestive of a biphasic disposition, with irreversible loss, of lactate from the horses.

Each of the points motivates the utilization of kinetic modeling software (21) to help explore these responses.

### Modeling the Lactate Disposition

There are now three relatively common approaches to building and using pharmacokinetic kinetic (PK) models to explore systems: 1) Gabrielsson and Weiner’s (22) approach uses clearances (within the system), volumes of distribution, and drug or metabolite blood levels to fabricate accounts of systems for an array of challenging and significant reasons. While the basis for this approach is undeniably sound, the manipulation of this subset of modeling objects can seem quite foreign to the PK investigator [Pharmacokinetic: meaning the study of the time course of drug or metabolite concentrations in different body spaces, e.g. blood, plasma, cerebrospinal fluid, and tissues (22)], and hence may quite likely not be readily embraced.

2) A strong case seems to have been made by Rowlands and Tozer (23) for the advantages offered by the ubiquitous Macro Constant model. Their reason for promoting this line of investigation is essentially linked to the principle that all of the information in linear PK data is actually encapsulated in the indices, A, alpha, B, beta etc., of the Macro Models. Thus, creative use can be advanced by accessing this information as a tool for extending our third model structures, 3) Micro Rate Models. We have actually developed a novel approach to enhancing Micro Rate model data using a form of Kinetic Imputation. Here, using modeling software (21) it is possible to extend the kinetic data using added time predictions based on the Macro model. Our reason though, in promoting Micro Rate constant models, is because they are susceptible to manipulation of the model topology (inputs, outputs, and exchanges) to meet the needs of, otherwise unavailable, approaches, without disrupting the system eigenvalues.

Throughout this report we will be referring to Micro Rate constant models and these will be solved, and fitted to data using the WinSAAM modeling software, see **Supplementary Data 1** (essentially, explaining the WinSAAM syntax) and, an allied account, **Supplementary Data 2** (for a breakdown of the semantics of the WinSAAM modeling elements used in this investigation). Finally, **Supplementary Data 3** outlines the critical elements in the models used and the manipulation of their units.

### The Units of Models and Modeling Objects

There are essentially three layers of modeling elements and their units falling under the investigator’s control for the manipulation of the system. The first is the system inputs, e.g. infusions, and allied external controls, the second relates to the internal modeling objects impacting the various determinations called for by the system’s investigation, and, finally, the third layer of manipulations amounts to transforming our intermediate determinations from the second layer to match the requirements of the external objects. The last array of objects usually serve as precursors to data or measurements reflecting the rationale for our investigation.

Consider the lactate infusion administered in this study. Setting up the infusion calls for selection of its units e.g. mmol.min^-1^, or mmol.kg^-1^.min^-1^. Motivating our choice here could be susceptible to a) keeping numbers manageably small, or b) simplifying the algebra associated with the second level of our processing, or c) ensuring that the unit choice blends our analysis to be ready for the final, third, step in preparing for the observation, or measurement, units.

There is also some back-wash from our units. The units of any object do not exist in isolation. Each object abuts other objects and these interfaces need to match one another unit-wise or take on a critical, possibly final, step in a chain, gradually assuring completeness in the end.

We consider an example: assume that we allow the unit of the infusion to be mmol.kg^-1^.min^-1^.

So long as the responses and inputs are linear we can write [see Common equations, **Supplementary Data 2** and (24)]

(1) UF1=L(1,2).F2−L(2,1).F1−L(0,1).F1+G1+G2

(2) F2`=L(2,1).F1−L(1,2).F2

See (**Supplementary Data, 1, 2 **and **3**) for an account of the nomenclature.

Here we will breakdown the equations to illustrate how the units of our infusion impact our state variables (F1, and F2). Since UF1 is the net rate of accumulation of lactate in compartment 1, L (1,2).F2 is the fractional rate of return of lactate from compartment 2 (F2) back into F1, and L (2,1).F1 is the fractional rate of movement of lactate from compartment 1 to compartment 2. G1 and G2 are inputs, G1, from the lactate infusion, and G2 from metabolism. If, as specified, our infusion is in the units of mmol.kg^-1^.min^-1^ then UF1 must also be of those units. Note that UF1 is a rate whereas F1, and F2 are amounts with the units of mmol.kg^-1^. The solutions to eq (1) and eq (2) are obtained by numerical integration from the modeling software.

Note from (**Supplementary Data 1** and **Supplementary Data 2**) the units of L (1,2), L (2,1), and L (0,1) are min^-1^ and their contexts are as fractional rates. Since these equations are linear all additive terms have the same units, and, of course, L (2,1).F1 and L (1,2).F2 have the same units, as well as do, G1 and G2 (mmol.kg^-1^.min^-1^).

To ease the ease the understanding of the two equations the reader is referred to **Supplementary Data 1** and **Supplementary Data 2**.

### Portraying Lactate Infusions

There have been considerable variations in the modeling of infusions explored over the years, and this seems to have emanated from the confidence investigators have had in their infusion pumps, or, in other mechanical devices to help administer infusions both completely, and smoothly. For example, one early approach, was to simply assert that infusions ran for the time intended (e.g. 15 minutes, in our case), and, in so doing, delivered the entire infusate within this allotted time (25–27). Other procedures (26) take a somewhat more realistic approach allowing the infusion to start slowly, gather speed, and then slow down again towards their climax (i.e. a sort of rhomboidal pattern of delivery and passage).

We have considered a novel approach here in which the speed of infusion delivery and the duration of delivery are each sensed and estimated using a multi-cell delay system which turns the infusion off soon after detecting that the administration of the content is reached. Since our model does not rely on any assumptions in regards to the state of perturbation of the system, or in other words, whether the system is in steady state or not, it is equally applicable in both states.

Indeed, we propose to examine the responses of three infusion delivery systems on aspects of the lactate kinetics as follows: A Simple Model (S) using rigid modeling tools to confer infusion design and limitations (e.g. infusion duration and amount) on the infusion units (e.g. mmol.min^-1^), A Unit Model (U) with the same infusion machinery as the S model but allowing the infusion units to maintain those of the study design (i.e. mmol.kg^-1^.min^-1^, in our case), a Novel Model (N) using the software delay machinery (21) to automatically detect completeness of the infusion delivery, and the infusion duration. The N model will use the same units as those for the U model.

To ensure that there is no disruption from the novel infusion machinery we simultaneously evaluated several of the common PK dependencies discussed by Gabrielsson and Weiner (22), (e.g. Volumes of distribution, Clearance rates, Macro rate constants, and their half-lives, along with others) using both the U model and the N model. Then we ran concordance tests (28) to confirm that the pattern of dependencies among the N model calculations were not significantly different, or divergent, from those of the U model (which we recall used simplified methods for infusion modeling). To confirm the differential equation solution estimates of the dependencies we also calculated these using the WinSAAM matrix equation facility where appropriate.

Finally, to emphasize the significance of the basal lactate level in regard to its prominence in our kinetic analysis, and, to recognize that it was based on an average of many more (13, typically) observations we used Bayesian methods here (21, 24, 29) based on the distribution of the basal lactate values.

## Results

Five horses successfully completed the lactate infusion (20) and we compared the stability of the common indices of the PK disposition among some of these to judge the consistency of our results across the study.

In our **Supplementary Data 1** and **Supplementary Data 2**, we provide a comprehensive guide to the common indices, or model elements, that we intend to investigate in regard to their susceptibility to vary in association with our choice among the three lactate infusion models (S, U, and N).

In **Table 1** and **Figure 2** we present the macro constant models for each horse with dispositions displayed. An outstanding feature of the table, and these plots, is the very stable estimates of the Macro constants in spite of the quite substantial range in horse weights.

**Table 1:** Independently estimated macro constants and their standard errors are shown along with the respective horse weights.

Horse	A	CV	a	CV	B	CV	b	CV	Weight [kg]
1	0.694	13%	0.247	30%	1.492	5%	0.008	17%	484
2	1.041	12%	0.092	23%	0.735	17%	0.002	74%	588
3	0.738	10%	0.101	16%	1.104	7%	0.006	17%	450
4	0.604	7%	0.269	17%	1.183	3%	0.007	8%	503
5	0.698	16%	0.202	35%	1.337	8%	0.011	19%	480

**Figure 3:**
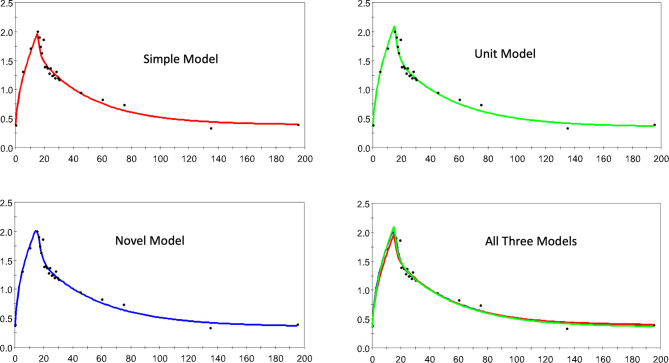
The estimated and observed lactate disposition for the S, U, and N models separately, and all models together are shown for horse 5. Note uniform offset to pre-infusion mean lactate level.

**Figure 3** shows 4 dispositions plots for horse 5 with the upper left relating to the S model, and upper right relating to the U model, lower left to the N model and lower right all three models.

**Figure 4:**
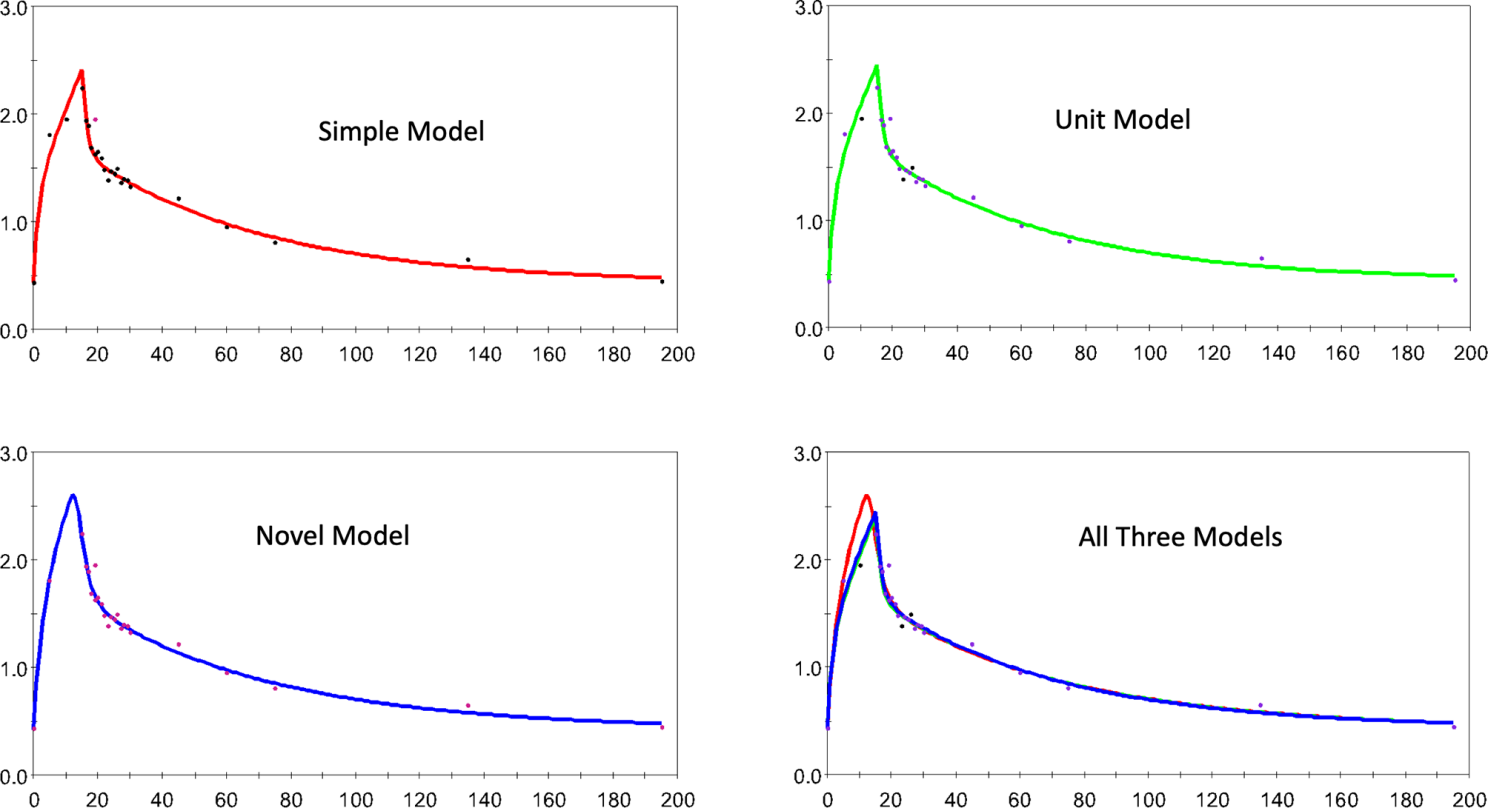
The estimated and observed lactate disposition for the S, U, and N models separately, and all models together are shown for horse 1. Note uniform offset to pre-infusion mean lactate level.

**Figure 4** a similar collection of plots but for horse 1 here.

**Figure 5:**
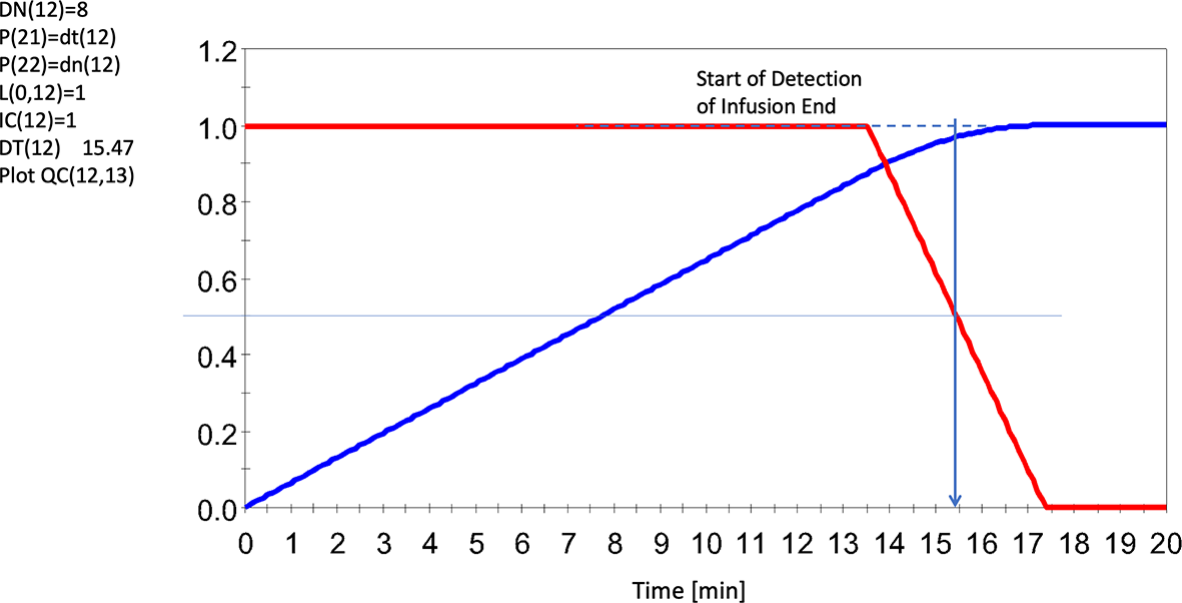
Sensing the End of the Infusion Using an 8 Cell Delay System (red line), and accumulated input lactate infusion UF (13) = 1.F (12)/DT (12) (blue line). Note dashed line regarding agreements of results. Note this infusion seemed to run for slightly over 15 mins (albeit slowly here) whereas the average for all infusions was ~14 min. Red line represents the solution to F (12). Horse 5 shown here. All levels were scaled to invoke generalizability. For further details, see **Supplementary Data 2** and **Supplementary Data 3**.

In **Figure 5** we demonstrate how the N model, applied to the infusion for horse 5, is able to detect the completion of the infusion allowing isolation of duration and net lactate administered.

**Table 2** presents the final estimates, their errors, for the adjustable parameters for horse 5, and for each of the 3 models explored. It is quite clear that in spite of the differences in numbers of adjustable parameters there is very little change in parameter value estimates by model form (S, U, or N).

**Table 2:** The estimates of the adjustable parameters (and their errors) for horse 5 using the SU, and N models (top to bottom).

PARAMETER	VALUE	ERROR	CV
Simple Model			
P (1, 0)	0.419	0.020	5%
K (1, 0)	0.009	0.001	6%
L (0, 1)	0.072	0.003	5%
L (2, 1)	0.280	0.013	5%
L (1, 2)	0.189	0.012	7%
Unit Model			
P (1, 0)	0.357	0.003	1%
P (2, 0)	0.215	0.007	3%
L (0, 1)	0.070	0.002	3%
L (2, 1)	0.209	0.014	7%
L (1, 2)	0.137	0.005	3%
Novel Model			
DT (12, 0)	15.400	0.187	1%
P (1, 0)	0.356	0.003	1%
P (2, 0)	0.181	0.014	8%
L (0, 1)	0.081	0.006	7%
L (2, 1)	0.266	0.034	13%
L (1, 2)	0.133	0.005	4%

P (1) is the mean pre-infusion baseline (mmol.kg^-1^). K (1) is the inverse of the lactate pool size (L) (See **Supplementary Data 2** and **Supplementary Data 3** for model S). P (2) is the lactate pool size (L.kg^-1^) (See **Supplementary Data 2** and **Supplementary Data 3** for model U and N). L(I,J) are fractional transfer rates (min^-1^) (See **Supplementary Data 1**). Please ignore the zeros (‘0’) in the right subscript. That is, for example, P (1,0) = P (1).

**Figure 6:**
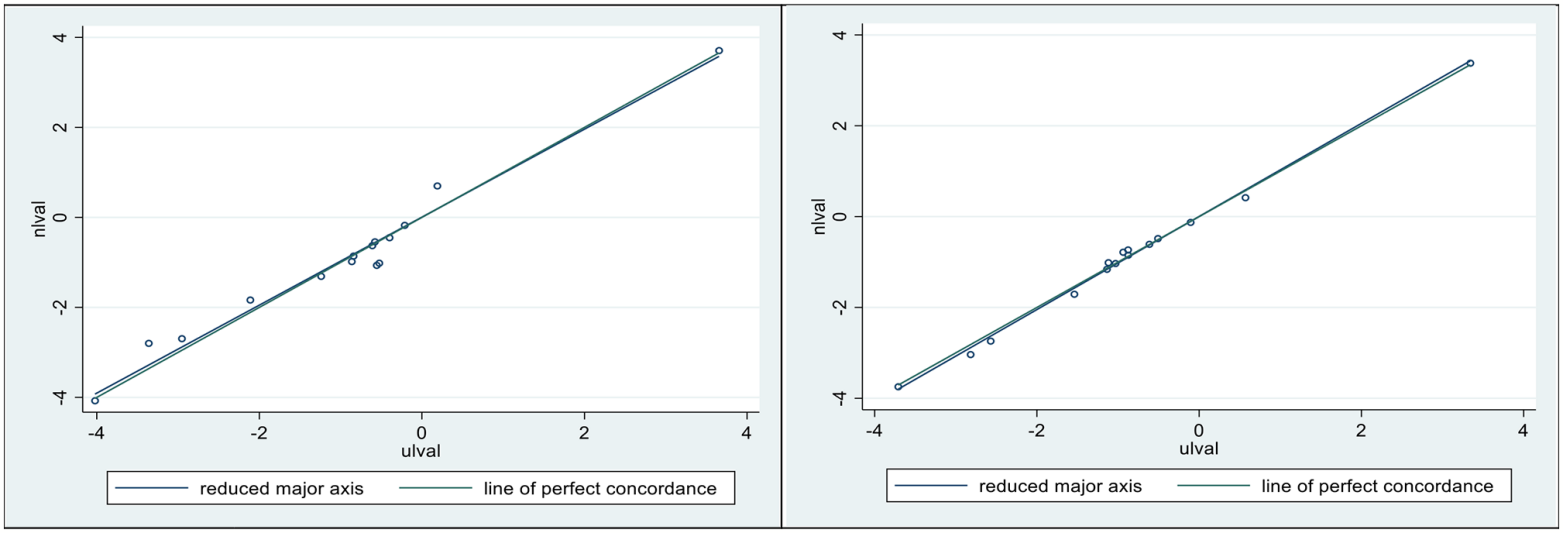
Lin’s concordance correlation coefficient (CCC) was 0.98 ± 0.05 for horse 1 (Left), and 0.997 ± 0.001 for horse 5 (Right). Plotted sloping lines represent estimated concordances (identified as the reduced major axis) and a line of perfect concordance, passing, as nearly as possible, through all pairs of observations.

In **Figure 6**, we present concordance plots (and measures) for horses 1 (left) and 5 (right), using their respective dependencies. In each of these plots the N model (is the vertical axis) and the U model (the horizontal axis). The goal here was to determine how well the dependencies were preserved in regard to the respective infusion models. The values for the concordances (28, 30) were 0.980 ( ± 0.05) for horse 1, and 0.997 ( ± 0.001) for horse 5.

## Discussion

Bearing in mind the array of complicated decisions investigators need to negotiate as they prepare for the kinetic analysis of challenge data we have here explored two critical questions. First, is there a convenient and consistent way of managing the units of the information that comes from challenge studies? Second and final, could there be a way of assessing the implication of system infusions lending itself to tracking and compensating for unfortunate issues that arise in association with this type of challenge?

To address these questions, we mounted a series of partial modeling approaches: indeed, we proposed three models to help us here, a Simple Model (S), a Unit Model (U), and a Novel model (N). The S model took a path enabling us to accurately and systematically build a model of the system (infusion, mixing and clearance, for example) using as many of the prefabricated modeling elements as the simulation and analysis exercise called for. The U model followed the S model in regard to appropriating software tools as called for by the modeling purpose but it deviated from the S model when it came to specifying the units of the modeling objects. Most significantly here, the U model called for specification of the units of the investigation to be created around the units of the infusion, in our case mmol.kg^-1^ (or mmol.kg^-1^.min^-1^). This single maneuver made it extremely easy to specify the array of units for all objects in the model, and to perform verifiable steps in possibly implicating dependencies.

But units were just one of the issues we intended to address, the other was to see if our N model (with Novel approaches to tracking infusions) was able to help us to discover 1) whether we could capitalize on the modeling software, this time allowing the manipulation of a multicell delay detection system enabling us to automatically find the best guides as to what transpired as our infusion advanced. And, 2) at what cost would this type of service present in regard to offsetting (possibly corrupting) the evaluation of common pharmacokinetic dependencies (22) as a potential collateral consequence of their operation. Armed with 15 common dependencies and our U and N models we were able to present preliminary evidence that the invocation of the delay machinery presented relatively few adverse consequences for these determinations. Indeed, the concordance of dependencies between the U and N-based models revealed that there were minimal corrupting side effects emanating within the N system.

## Conclusion

Problems with infusion pumps are ubiquitous, and it is far from commonly an operator error leading to this situation. It can be extremes in fluid viscosity and/or heterogeneity, and clumps among the infusate. Indeed, based on one experimental failure (20) (one of our 6 original horses had to be withdrawn due to pump problems), this report was undertaken to explore the possibility of software modeling tools providing quantitative backup of our administration efforts. We cannot categorically state that we have the solution to the issue at this point, but we do believe that we have created a case for at least considering exploring our ideas, and that the more investigators try the methods discussed in this paper the stronger may be the information assembling to endorse this style of operation.

Besides providing a backup for the clinical investigators in terms of administration efforts outlined above, our methodologies offer several additional clinical benefits. First, modeling infusions using the actual units in which the infusion was administered leads to the accurate and straightforward specification of all subsequent system elements from within our PK account. Second and final, the current lactate kinetic models offer a better understanding of how a possible plasma increase in lactate can be attributed to increased production and the extent to which it results from a change in the kinetics of lactate.

One single and clear-cut recommendation we can offer though, is that modeling infusions using the actual units in which the infusion was administered leads to the simple and accurate specification of all subsequent system elements from within our PK account.

## Data Availability Statement

The raw data supporting the conclusions of this article will be made available by the authors, without undue reservation.

## Ethics Statement

The animal study was reviewed and approved by the Institutional Animal Care and Use Committee (IACUC) of the University of Illinois at Urbana-Champagne.

## Author Contributions

DS reviewed and edited the text. PAW conducted the experimental study, reviewed the text. RCB conducted the analysis, wrote, reviewed, edited the article, and produced the figures. All authors contributed to the article and approved the submitted version.

## Conflict of Interest

The authors declare that the research was conducted in the absence of any commercial or financial relationships that could be construed as a potential conflict of interest.
