# Supplementary material for: Modeling Challenge Data to Quantify Endogenous Lactate Production
Source: Front Endocrinol (Lausanne). 2021 Jun 28;12:656054. doi: 10.3389/fendo.2021.656054 (PMC8277460; doi:10.3389/fendo.2021.656054)
Supplement: Supplementary file 1 [file DataSheet_1.docx]

Supplemental Data 1. WinSAAM Syntax

The major emphasis for the Supplementary Data shown here is to highlight the common modeling objects available in WinSAAM and to illustrate their context as we negotiate the 3 distinct models explored in our report.

Three models are discussed here (see suffixes), S (Simple Model), U (Unit Model), and N (Novel Model). Different forms of the state variables (F(J)), and different scaling factors (e.g. P(2), for U and N, and K(1)^-1^, for S) need to be applied to bring the Units of F(J) to the same as those of QO(J) (i.e. mmole.L^-1^). When scaling is applied to F(J) the resultant calculations are referred to as QC(J) values

^#^Delay abruptness is usually identified as the half-width (for DN(J)=2) of the delay (i.e. DT(J)/2) but for DN(J)=4 the halfwidth is reduced to half of this value i.e. DT(J)/4.

| **WinSAAM Object** | **Class** | **Unit** | **Context** |
| --- | --- | --- | --- |
| F(J) | Solution to state equation (for component J | *[mmole]^s^ or [mmole.kg^-1^]^U,N^ | Predictions of Lactate observations |
| UF(J) | Derivative of F(J) or Input function for F(J) to generalize inputs | *Rate of lactate accumulation in comp J [mmole.min^-1^]S or [mmole.kg^-1^.min^-1^]^U,N^ | Net rate of lactate appearance in compartment J |
| L(J,I), or L(0,1) | Fractional transfer from compartment I to compartment J, or Irreversible fractional removal rate from compartment 1 (L(0,1)) | [min^-1^] | Expressing state equations for lactate movement |
| IC(J) | Initial condition of F(J) (Observed) | [mmole.L^-1^] | Reference for lactate as start of infusion |
| K(J) | Multiplicative scale factor for component J | [L^-1^]^S^ | *Volume of distribution, Vd^S^ |
| G(J) | Time dependent function, with index J, holding equation elements for UF(J) | Rate of lactate input to UF(J) with units of UF(J) |  |
| P(J) | Internal Parameter with index J holding estimated values for pool sizes | Depending on internal calculation [L.kg^-1^] | *Volume of distribution, Vd^U,N^_,_ as P(2), say |
| DT(J) | A relative delay in the flow thru aspects of the system | Time [min] | Impeding movement or detecting an unchanged state |
| DN(J) | The number of cells used to set the ^#^abruptness of a delay | No units, just a number e.g. DN(2)=8 | If a delay is assigned a cell number of 4 it will lead to a delay portrayal with twice the abruptness of a delay with 2 cells |
| QC(J) | Converted from F(J) to Units of Observations for U and N models | [mmole.L^-1^] | Change state variables from natural form to estimates of QO(J) |
| QO(J) | Observations associated with compartment J | [mmole.L^-1^] | Iteratively adjusting model parameters (e.g. L(I,J), or K(J)^S^ for example) to bring QC(J) to best fitting QO(J) |
