# Supplementary material for: Modeling Challenge Data to Quantify Endogenous Lactate Production
Source: Front Endocrinol (Lausanne). 2021 Jun 28;12:656054. doi: 10.3389/fendo.2021.656054 (PMC8277460; doi:10.3389/fendo.2021.656054)
Supplement: Supplementary file 2 [file DataSheet_2.docx]

Supplemental Data 2. Modeling Elements

There are three sections to this Figure: A) The model graphic, reflecting the elements (see SD1) of the system used in our

investigation. B) The common set of differential equations used to solve our model and fit the specific adjustable parameters to the S (simple), U (unit), and N (novel) models. C) the conversion of the internal units to enable their reconciliation with the observation units (Note that the S model could be fitted from the state variable level without any need to transform the internal units).
