# Supplementary material for: Modeling Challenge Data to Quantify Endogenous Lactate Production
Source: Front Endocrinol (Lausanne). 2021 Jun 28;12:656054. doi: 10.3389/fendo.2021.656054 (PMC8277460; doi:10.3389/fendo.2021.656054)
Supplement: Supplementary file 3 [file DataSheet_3.docx]

Supplemental Data 3. Critical Elements in the Models

For reader convenience we summarize the critical elements in the S, U, and N models.

We remind the readers that we would be obliged to provide any help (email: [drrayboston@yahoo.com](mailto:drrayboston@yahoo.com); sdarko@upenn.edu) they would like in implementing the models discussed in this paper, regardless of the modeling software they use.

P1=Basal Lactate [mmole.L^-1^] S U and N models

P2=Vd [L.kg^-1^] U model and N model

P13=1/K1=Vd [L] S model

F12=1 if 0<=t<=15 S and U model

Dose=1 [mmole.kg^-1^]

G1=Dose.F12.Wgt/15 [mmol.kg^-1^].F12.[kg][min^-1^] =[mmol.min^-1^] S model

G2=p1.l(0,1).p(13) [mmole.L^-1^].[min^-1^].[L] = [mmol.min^-1^] S model

G3=F1.[K1^-1^] [mmole.L^-1^] S model

G1=Dose.F12./15 [mmole.kg^-1^].F12.min^-1^=[mmole.kg^-1^min^-1^] U model

G2=p1.l(0,1).p2 [mmole.L^-1^][min^-1^]L.kg^-1^]=[mmole.kg^-1^min^-1^] U model

G3=F1/P2 [mmole.kg^-1^][L.kg^-1^]^-1^=[mmole.L^-1^] U model

G1=Dose.F12*/DT12 [mmole.kg^-1^].F12.[min^-1^]=mmole/kg/min] N model F* is sensing tool

G2=p1.l(0,1).p2 [mmole.L^-1^].[min^-1^].[L/kg]=[mmole/kg/min] N model

G3=F1/p2 [mmole.kg^-1^]/[L.kg^-1^]=[mmole.L^-1^] N model

G3 converts modeling objects from internal units to external, or observation, units [mmole/L]

Note: F12, for the S and U models is a rectangular function equal to 1 if 0<=t<=15 min

WinSAAM Specification of Infusion into system via F12 and DT12

Note: F*12, for the N model is the response of DT12 where DT12 is adjustable .. viz:

Dt(12) 15 100

DN(12)=8

IC(12)=1

L(0,12)=1
